# Vitamin A Protects the Preterm Lamb Diaphragm Against Adverse Effects of Mechanical Ventilation

**DOI:** 10.3389/fphys.2018.01119

**Published:** 2018-08-13

**Authors:** Yong Song, MarJanna Dahl, Wendy Leavitt, Jeremy Alvord, Calan Y. Bradford, Kurt H. Albertine, J. Jane Pillow

**Affiliations:** ^1^School of Human Sciences, The University of Western Australia, Crawley, WA, Australia; ^2^Centre for Neonatal Research and Education, Division of Paediatrics and Child Health, Medical School, The University of Western Australia, Crawley, WA, Australia; ^3^School of Public Health, Curtin University, Bentley, WA, Australia; ^4^Centre for Genetic Origins of Health and Disease, The University of Western Australia, Curtin University, Crawley, WA, Australia; ^5^Department of Pediatrics, University of Utah, Salt Lake City, UT, United States

**Keywords:** ventilator-induced diaphragm dysfunction, VIDD, bronchopulmonary dysplasia, BPD, disuse atrophy, retinoids, retinol

## Abstract

**Background:** Preterm infants are deficient in vitamin A, which is essential for growth and development of the diaphragm. Preterm infants often require mechanical ventilation (MV) for respiratory distress. In adults, MV is associated with the development of ventilation-induced diaphragm dysfunction and difficulty weaning from the ventilator. We assessed the impact of MV on the preterm diaphragm and the protective effect of vitamin A during MV.

**Methods:** Preterm lambs delivered operatively at ∼131 days gestation (full gestation: 150 days) received respiratory support by synchronized intermittent mandatory ventilation for 3 days. Lambs in the treated group received daily (24 h) enteral doses of 2500 IU/kg/day vitamin A combined with 250 IU/kg/day retinoic acid (VARA) during MV, while MV control lambs received saline. Unventilated fetal reference lambs were euthanized at birth, without being allowed to breathe. The fetal diaphragm was collected to quantify mRNA levels of myosin heavy chain (MHC) isoforms, atrophy genes, antioxidant genes, and pro-inflammatory genes; to determine ubiquitin proteasome pathway activity; to measure the abundance of protein carbonyl, and to investigate metabolic signaling.

**Results:** Postnatal MV significantly decreased expression level of the neonatal MHC gene but increased expression level of MHC IIx mRNA level (*p* < 0.05). Proteasome activity increased after 3 days MV, accompanied by increased *MuRF1* mRNA level and accumulated protein carbonyl abundance. VARA supplementation decreased proteasome activity and FOXO1 signaling, down-regulated *MuRF1* expression, and reduced reactive oxidant production.

**Conclusion:** These findings suggest that 3 days of MV results in abnormal myofibrillar composition, activation of the proteolytic pathway, and oxidative injury of diaphragms in mechanically ventilated preterm lambs. Daily enteral VARA protects the preterm diaphragm from these adverse effects.

## Background

Mechanical ventilation (MV) is a lifesaving supportive management approach for patients with acute respiratory failure. However, prolonged MV decreases the force-generating capacity of the diaphragm, known as ventilator-induced diaphragmatic dysfunction (VIDD). VIDD increases the risk of difficulty in weaning patients off ventilator support (Vassilakopoulos and Petrof, 2004). The effects of MV on muscle function are well studied in adult animal models. However, whilst the underlying mechanisms are incompletely understood, VIDD is associated with muscle fiber remodeling, abnormal diaphragmatic protein turnover (decreased protein synthesis, increased proteolysis, or both), and oxidative injury (Shanely et al., 2002, 2004; Powers et al., 2009, 2013).

Preterm infants have reduced diaphragm contractility compared to their term gestation counterparts (Dimitriou et al., 2003; Lavin et al., 2013). Diaphragm fiber type analysis across a number of species indicates a low content of slow Type I (fatigue resistant) muscle fibers in the immature diaphragm (<10% at birth) versus a higher content in the mature diaphragm (∼50–60%) (Keens et al., 1978; Maxwell et al., 1989). In humans, adult proportions of Type I fibers are not attained until 1 y of postnatal age (Keens et al., 1978). Neonatal rat diaphragm has a high proportion of fast Type II fibers, which is more susceptible than Type I fibers to muscle atrophy driven by catabolic signaling (Dekhuijzen et al., 1995). Additionally our previous data show a significant increase in reactive oxygen species (ROS) production in lamb diaphragm on initiation of air breathing (Song and Pillow, 2013b): the increase in ROS is likely a result of exposure to higher oxygen concentration than experienced in the hypoxic *in utero* environment, and immature development of the antioxidant defensive system. Therefore, preterm infants may be more susceptible to VIDD compared with term or adult subjects, owing to differences in fiber-type composition (Keens et al., 1978; Lavin et al., 2013) and oxidative capacity (Song and Pillow, 2013b).

Retinoids are important regulators of differentiation and cell proliferation and play a fundamental role in diaphragm development. Retinoic acid (RA), an irreversibly oxidized form of vitamin A (retinol), has important hormone-like growth factor effect on tissues, including muscles. Vitamin A deficiency decreases the rate of protein synthesis in rat skeletal muscle (Narbonne et al., 1978). Vitamin A deficiency also alters expression of contractile proteins (Downie et al., 2005), increasing expression of fast myosin heavy chain (MHC) isoforms by 800% in preterm neonates, and paradoxically, decreasing fast myosin by 50% in one day old neonates (Downie et al., 2005). An increase in fast twitch characteristics of the diaphragm of preterm neonates could make the diaphragm more susceptible to fatigue and prone to contraction-induced damage and dysfunction. Importantly, vitamin A deficiency also contributes to development of bronchopulmonary dysplasia (BPD), the chronic respiratory impairment associated with preterm birth, and failed alveolarisation, the incidence of which is linked to duration of MV (Darlow and Graham, 2011). Vitamin A supplementation reduces the prevalence of BPD (Darlow and Graham, 2011). The association of deficiency of vitamin A and RA with impaired growth and development of both the diaphragm and the lung highlights these molecules as potential options for therapeutic and preventive intervention to reduce the severity of diaphragmatic and respiratory impairment in preterm infants.

We hypothesized that the diaphragm of preterm neonates was susceptible to dysfunction associated with MV. We further hypothesized that supplementation of ventilated preterm lambs with a combination of vitamin A and all-*trans* retinoic acid (VARA) would counteract the detrimental effects of MV on the diaphragm and thereby reduce the severity of VIDD. Therefore, the present study aimed to establish the impact of MV on fiber composition, protein signaling, proteolytic activity, and tissue oxidation of preterm lambs, and to identify the impact of enteral VARA on these outcomes.

## Materials and Methods

### Animal Management and Study Design

The animal experiments were approved by the Institutional Animal Care and Use Committee at the University of Utah, Health Sciences Center (IACUC11-11002). Breeding, ewe-management and postnatal lamb ventilation followed protocols described previously. (Reyburn et al., 2008; Albertine et al., 2010; Joss-Moore et al., 2016) Briefly, time-mated ewes received intramuscular antenatal dexamethasone (6 mg; Vedco, Inc., St. Joseph, MO, United States) 24 h prior to planned surgical delivery at 130–132 days of gestation (term∼150 days). Preterm lambs were assigned randomly to four groups: (1) Fetal Start (130–132 days) and (2) Fetal End (133–135 days), (3) MV; (4) MV with Aquasol A palmitate 2500 IU/kg/day (Pfizer, United States) and all-*trans* retinoic acid 250 IU/kg/day (MV + VARA). VARA was diluted in canola oil and given orogastrically, beginning immediately after delivery and repeated at 24 h intervals. Plasma retinol was measured by quantitative HPLC (ARUP Lab, Salt Lake City, UT, United States).

Preterm fetal lambs were killed (Beuthanasia solution 100 mg/kg, i.v., Schering-Plough Animal Health Corporation, Union, NJ, United States) either immediately on exteriorisation prior to commencement of breathing (fetal reference groups one and two) or after 3 days of postnatal management and MV as described below (groups three and four). The left costal hemidiaphragm was harvested immediately after euthanasia and snap frozen (-80°C) for biochemical and molecular experiments (Song et al., 2013a).

### Mechanical Ventilation Protocol

Preterm lambs in the MV and MV + VARA groups were intubated, using a cuffed endotracheal tube (3.5–4.0 French) through which 10 mL of lung liquid was aspirated and replaced with beractant (4 mL, NDC 0074-1040-08; Ross Products Division, Abbott Laboratories, Columbus, OH, United States). Each fetus was then removed from the uterus before its umbilical cord was ligated and cut.

Preterm lambs were managed by pressure-limited, synchronized intermittent mandatory ventilation, with warmed and humidified 100% oxygen (Bird VIP ventilator, model 15215; Bird Products Corporation, CA, United States). Arterial blood gasses, pH, electrolytes, and glucose were measured hourly, using an indwelling arterial catheter in the right common carotid artery. Oxygen saturation was monitored by pulse oximetry (target was 90–94%). Vascular pressures and heart rate were recorded continuously (model V6400; SurgiVet, Waukesha, WI, United States). Dextrose was infused intravenously to maintain plasma glucose level between 60 and 90 mg/dl. Plasma concentrations of total protein and hematocrit were measured at 6 h intervals. Lambs received intermittent postnatal sedation, postnatal antibiotics and graduated incremental enteral feeding as tolerated, in accordance with previous studies (Joss-Moore et al., 2016).

Initial respiratory rate was 60 breaths/min and inspiratory time was 0.3 s. Peak inspiratory pressure (PIP) was adjusted to attain a target *P*a_CO2_ between 45 and 60 mm Hg and pH between 7.25 and 7.35. Positive end-expiratory pressure (PEEP) was initially set at 8 cm H_2_O. Target expiratory tidal volume, measured by the ventilator, was 5–7 mL/kg/breath. The concentration of inspired O_2_ was adjusted to target a *P*a_O2_ between 60 and 80 mm Hg. Target Pa_O2_ and Pa_CO2_ were reached within 1–2 h of postnatal life. All of the preterm lambs received an intravenous loading dose of caffeine citrate within 30 min of delivery to stimulate ventilatory drive (15 mg/kg, given over 2 h; Mead Johnson and Co., Evansville, IN, United States), followed by maintenance treatment with 5 mg/kg every 24 h (Joss-Moore et al., 2016).

### Isolation of mRNA and Quantitative Real Time RT-PCR

RNA purification, reverse transcription, and quantitative real time RT-PCR were performed (Song and Pillow, 2012). The primers of MHC gene (*MHC neonatal*, *MHC I*, *MHC IIa*, *MHC IIb*, and *MHC IIx*) were designed to target specific coding sequences of MHC isoforms (**Table 1**). The method of expression assay for the atrophic genes (*MAFbx* and *MuRF1*), cytokine genes (*IL-1*β and *IL-6*), antioxidant genes (*glutathione peroxidase 1*, *GPX1*; *superoxide dismutase 1*, *SOD1*, and *catalase*), as described previously (Song and Pillow, 2012; Song et al., 2013a,c). Fluorescence signal was analyzed and normalized against 18S RNA, which was constant amongst groups. Relative expression levels were calculated, using the 2^-ΔΔCT^ method. Results are presented as fold-change relative to the Fetal Start unventilated reference group.

**Table 1:** Primer sequences for MHC isoforms.

Gene	Primer sequence (5′-3′)	Accession No. (GenBank)	Product size (bp)
*MHC neonatal*	F CCTACTGCTTCGTGGCTGACT	XM_004012708	107
	R CACCAGCGTCCTGTTGTCT		
*MHC I*	F AGGACGTCTTTGTGCCTGATGA	XM_004010325	107
	R GGTCACTGTCTTGCCATGC		
*MHC IIa*	F ATCTGTCTTTGTGGCCGAGC	XM_004012707	106
	R TGTCAGAGTCGCCCCTCCT		
*MHC IIb*	F CAAAGAGAAGCATGTTATCTTTC	XM_004012705	161
	R TAGACGTGCCTTCTGGGCTGA		
*MHC IIx*	F TGGCCAGCAACATGGAGACT	XM_004012706	147
	R GACGTGCTCTCTGAGTTGTT		


*F, forward; R, reverse; MHC, myosin heavy chain.*

### Muscle Protein Extraction and Western Blot Analysis

Cytosolic and nuclear proteins were isolated, as described previously (Song and Pillow, 2013b). Protein concentration was measured, using the Bradford method (Sigma, Sydney, Australia). Twenty μg protein samples were resolved on 4–15% TGX Stain-Free gels (Bio-Rad, Gladesville, NSW, Australia) and protein was transferred onto nitrocellulose membrane, using a Trans Turbo Blot system (Bio-Rad). Western blotting was done with primary antibodies for phosphorylated (p)-Akt (Ser473) (9271), total Akt (9272), p-mTOR (Ser2448) (2971), total mTOR (2983), p-p70S6 Kinase (Thr389) (9234), total p70S6 Kinase (2708), p-4E-BP1 (Thr70) (9455), total 4E-BP1 (9452), FOXO1 (9454), and NF-κB p65 (3987) from Cell Signaling Technology (Carlsbad, CA, United States). The primary antibodies were dissolved in phosphate-buffered saline containing 0.1% Tween-20 (PBST) and 0.5% skimmed milk in 1:2000 dilutions and added to the membranes at 4°C overnight. Bound antibodies were detected, using anti rabbit immunoglobulin conjugated with horseradish peroxidase (7074, Cell Signaling Technology). The secondary antibody was used in 1:3000 in PBST with 0.5% skimmed milk and incubated with membranes at room temperature for 1 h. After adding a chemiluminescent substrate (Thermo Fisher Scientific, MA, United States), immunoreactive protein signals were detected and quantified, using ChemiDoc MP Imaging System (Bio-Rad). The activities of cytoplasmic signaling molecules are shown as phosphorylated/total protein ratio. The value for nuclear FOXO1 and NF-κB content was normalized to total nuclear protein content of the same lane on the blot.

### Biochemical Analysis of Proteasome Activity and Oxidative Stress

The chymotrypsin-like peptidase activity of the 20S proteasome was measured fluorometrically in crude extracts using a commercial kit (BML-AK740 assay kit, Enzo Life Sciences, NY, United States). Protein carbonyl level was measured in cell lysates, using Protein Carbonyl Colorimetric Assay Kit (Cayman Chemical, Ann Arbor, MI, United States).

### Data Analysis

SigmaPlot (version 12.0, Systat Software Inc, San Jose, CA, United States) was used for statistical analysis. Differences among multiple groups were assessed, using one-way ANOVA, with a Tukey honestly significant difference (HSD) test for *post hoc* analysis. Non-parametric data were analyzed, using ANOVA on ranks. The Pearson correlation index was calculated to determine association among different variables, using linear regression analysis. We assessed statistical significance at *p* < 0.05. Data are presented as mean (SD) or median (range). There were no differences in oxygenation (PaO_2_/FiO_2_ or oxygenation index (OI) despite initial increased PaO_2_ and mean airway pressure (MAP) in the VARA group (**Table 3**).

## Results

### Lamb Characteristics

Descriptive characteristics for each group are presented in **Table 2**. No statistically significant differences were detected for gestational age, body weight, and sex at birth amongst the groups. Final weight was not different between the MV and MV + VARA groups of preterm lambs. Mean (SD) plasma retinol was increased in the MV + VARA group [0.087 (0.019) mg/L] compared to the MV group [0.065 (0.006) mg/L] (*p* = 0.021).

**Table 2:** Descriptive characteristics for the lambs used in the experimental study.

Group	Fetal start	Fetal end	MV	MV + VARA
N	7	7	10	6
GA	130–132	135–136	130–132	130–132
Sex (M:F)	3:4	3:4	5:5	6:0
Ewe parity (Twin:Single)	7:0	2:5	4:6	3:3
Birth weight	3.58 (0.71)	3.02 (0.44)	3.95 (0.63)	4.16 (0.62)
Final weight	N/A	N/A	3.77 (0.74)	3.83 (0.64)


*GA, gestational age; N/A, not determined; MV, mechanical ventilation; VARA, vitamin A and all-*trans* retinoic acid.*

**Table 3:** Postnatal respiratory variables.

Variable	Time (h)	MV	MV + VARA	*p*-value
FiO_2_ (%)				
	24	27 (10)	37 (22)	0.152
	48	28 (9)	30 (7)	0.271
	72	28 (10)	32 (15)	0.938
PaO_2_ (mm Hg)				
	24	73 (11)	91 (25)^∗^	0.042
	48	84 (12)	84 (11)	0.699
	72	78 (11)	69 (9)	0.083
O_2_ Saturation (%)				
	24	95 (2)	95 (3)	0.855
	48	96 (2)	95 (2)	0.519
	72	95 (3)	94 (2)	0.250
PIP (cmH_2_O)				
	24	16 (3)	19 (5)	0.212
	48	19 (3)	21 (4)	0.206
	72	19 (3)	21 (3)	0.189
MAP (cmH_2_O)				
	24	11 (1)	13 (1)^∗^	0.025
	48	12 (1)	12 (2)	0.981
	72	12 (1)	12 (1)	0.895
pH				
	24	7.33 (0.07)	7.32 (0.04)	0.733
	48	7.32 (0.04)	7.29 (0.06)	0.171
	72	7.34 (0.08)	7.38 (0.09)	0.326
PaCO_2_ (mm Hg)				
	24	46 (6)	54 (13)	0.124
	48	51 (8)	55 (8)	0.438
	72	53 (6)	52 (14)	0.179
PaO_2_/FiO_2_				
	24	299 (80)	281 (139)	0.793
	48	348 (139)	339 (114)	0.437
	72	296 (96)	265 (161)	0.519
OI				
	24	2.46 (1.10)	3.18 (2.06)	0.313
	48	2.45 (1.30)	3.02 (0.78)	0.364
	72	2.79 (1.67)	3.52 (3.48)	0.606


*MV, mechanical ventilation; VARA, vitamin A all-*trans*-retinoic acid; FiO_2_, fractional inspired oxygen; PaO_2_, arterial partial pressure of oxygen; PIP, peak inspiratory pressure; MAP, mean airway pressure; PaCO_2_, arterial partial pressure of carbon dioxide; OI, oxygenation Index [FiO_2_ × 100 × MAP/(PaO_2_ × 1.36)]. Values are mean (SD) or median (IQR) for parametric and non-parametric variables, respectively.*

### MHC mRNA Levels

**FIGURE 1:**
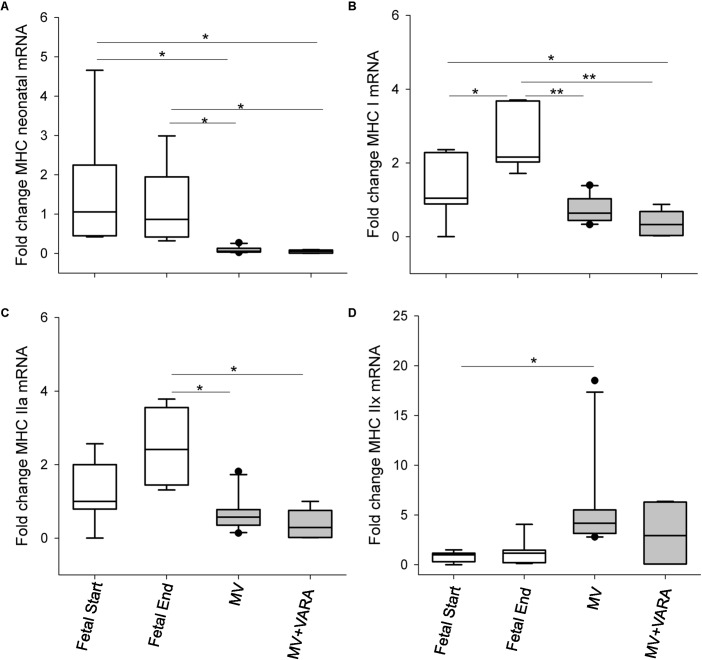
MHC molecules. Graphs show *MHC neonatal* **(A)**, *MHC I* **(B)**, *MHC IIa* **(C)**, and *MHC IIx* **(D)** mRNA levels in the groups of Fetal Start (*n* = 7), Fetal End (*n* = 7), MV (*n* = 10), and MV + VARA (*n* = 6). mRNA levels are expressed as fold-change relative to Fetal Start group. Boxes show are Median (25th and 75th centile), whiskers (error bars) show 10th, 90th centiles with solid circles showing outliers. ^∗^indicates *p* < 0.05, while ^∗∗^indicates *p* < 0.001. MHC, myosin heavy chain; MV, mechanical ventilation; VARA, vitamin A and all-*trans* retinoic acid.

Five MHC isoforms were quantified for differences in mRNA level in the diaphragm (**Figure 1**). Baseline *MHC IIb* mRNA level was very low and therefore its level was unreliable for quantification. Changes in MHC isoform mRNA levels were similar between the MV and MV + VARA groups. MV for 3 days reduced *MHC neonatal*, *MHC I*, and *MHC IIa* mRNA levels compared to unventilated Fetal Start and Fetal End reference groups, irrespective of VARA treatment. Conversely, 3 days of MV significantly increased mRNA level of *MHC IIx* in the MV group compared to both control groups.

### Protein Degradation

**FIGURE 2:**
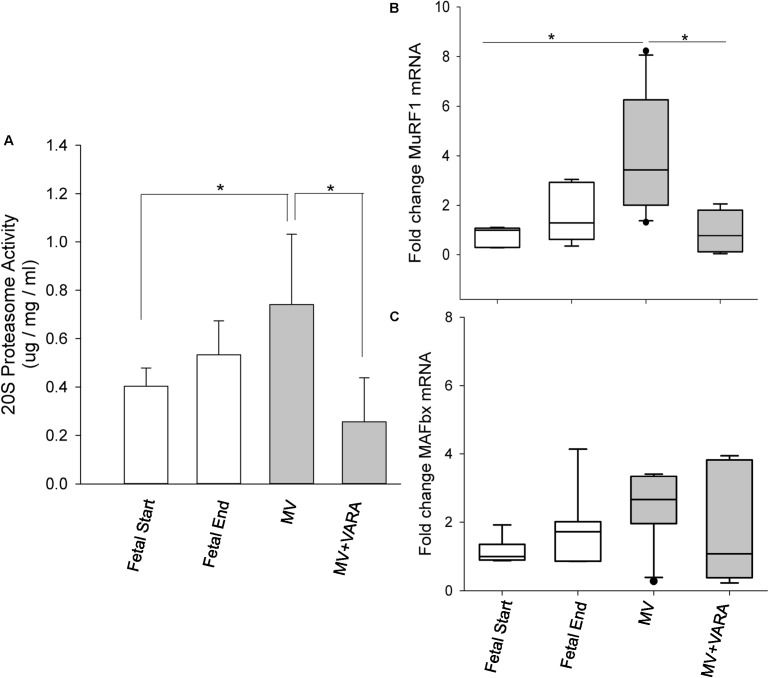
Proteolytic molecules. Graphs show 20S proteasome activity **(A)** and atrophic gene *MuRF1* **(B)** and *MAFbx* **(C)** mRNA levels in the groups of Fetal Start (*n* = 6/7), Fetal End (*n* = 6/7), MV (*n* = 9/10), and MV + VARA (*n* = 6). Values are Mean (SD) for 20S proteasome activity or Median (25th and 75th centile) for mRNA level data, expressed as fold-change relative to Fetal Start group. ^∗^indicates *p* < 0.05. MV, mechanical ventilation; VARA, vitamin A and all-*trans* retinoic acid.

The activity of the principal proteolytic pathway (ubiquitin proteasome pathway, UPP) was measured as a marker of proteolysis. Three days of MV significantly increased diaphragmatic level of UPP activity by 84% (**Figure 2A**). Daily VARA treatment significantly lowered proteolytic activity compared to the MV group (**Figure 2A**).

We also quantified the mRNA level of two key components of the 20S proteasome pathway (MuRF1 and MAFbx). *MuRF1* mRNA level increased significantly in the diaphragm of the MV group compared to both unventilated fetal reference groups (*p* < 0.05). Treatment with VARA prevented the MV induced increase in *MuRF1* mRNA level (*p* < 0.05, **Figure 2B**) to a level not different from either unventilated fetal gestational reference group. *MAFbx* mRNA level was not significantly different between the four experimental groups (**Figure 2C**), although the pattern of changes were the same as for *MuRF1*. As expected, UPP activity correlated significantly correlated with mRNA level of *MuRF1* (*r* = 0.785, *p* < 0.001, **Supplementary Data Sheet 1**).

### Molecular Signaling and Inflammatory Responses

We also investigated several key intracellular mediators of anabolic (Akt, mTOR, p70S6K, and 4E-BP1) and catabolic (FOXO1 and NF-κB) pathways to identify effects on signal transduction cascades.

**FIGURE 3:**
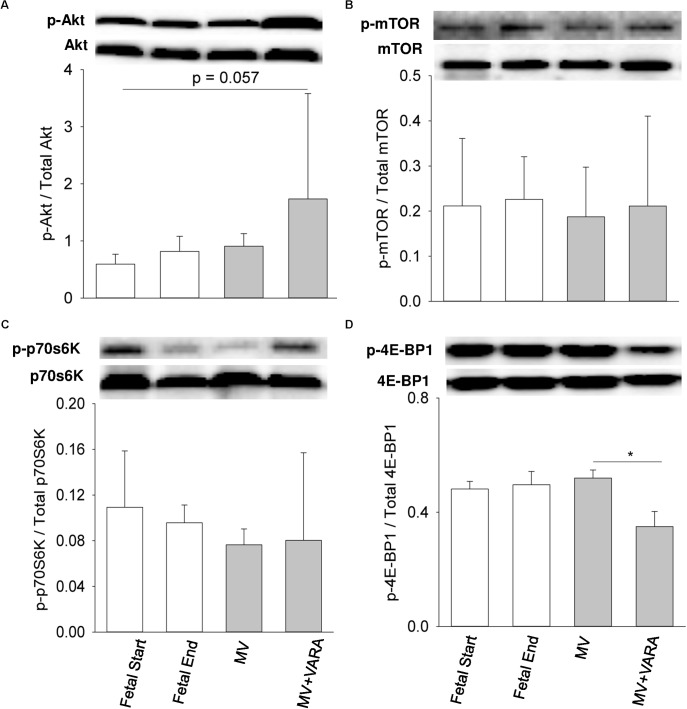
Cytosolic signaling molecules. Western blots illustrate protein abundance of signaling molecules in cytosolic fraction, using representative samples from each group above the graphs. Graphs show p-Akt/total Akt protein **(A)**, p-mTOR/total mTOR protein **(B)**, p-p70S6/ total p70S6 kinase **(C)**, and p-4E-BP1/total 4E-BP1 protein **(D)** in the groups of Fetal Start (*n* = 5), Fetal End (*n* = 5), MV (*n* = 5), and MV + VARA (*n* = 6). p: phosphorylated. Values are Mean (SD). ^∗^Indicates *p* < 0.05. MV, mechanical ventilation; VARA, vitamin A and all-*trans* retinoic acid.

Three days of MV alone did not change protein abundance in the diaphragm of any of the four measured anabolic pathway mediators (**Figure 3**). MV + VARA decreased the abundance of phosphorylated cytosolic 4E-BP1 compared to the MV group (**Figure 3D**). The level of phosphorylated cytosolic 4E-BP1 was not different among the MV+VARA and unventilated fetal reference groups.

**FIGURE 4:**
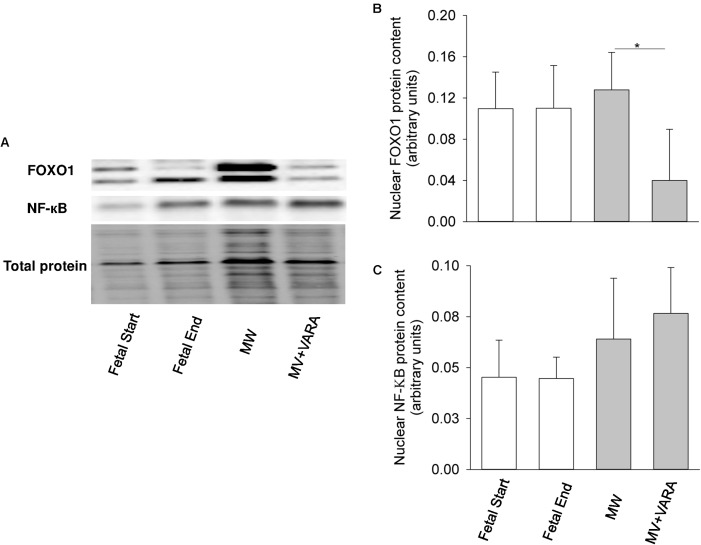
Nuclear FOXO1 and NF-κB. Western blots illustrate protein abundance of signaling molecules in nuclear fraction, using representative samples from each group **(A)**. Graphs show nuclear FOXO1 **(B)** and NF-κB **(C)** protein abundance in the groups of Fetal Start (*n* = 5), Fetal End (*n* = 5), MV (*n* = 5), and MV + VARA (*n* = 6). Values are Mean (SD). ^∗^indicates *p* < 0.05. MV, mechanical ventilation; VARA, vitamin A and all-*trans* retinoic acid.

For the two markers of catabolic pathways, neither nuclear FOXO1 nor NF-κB protein abundance were affected by 3 days of MV compared to both unventilated fetal reference groups (**Figure 4**). Protein abundance of nuclear FOXO1 was reduced in the MV + VARA group compared to the MV group (*p* < 0.05, **Figure 4B**).

**FIGURE 5:**
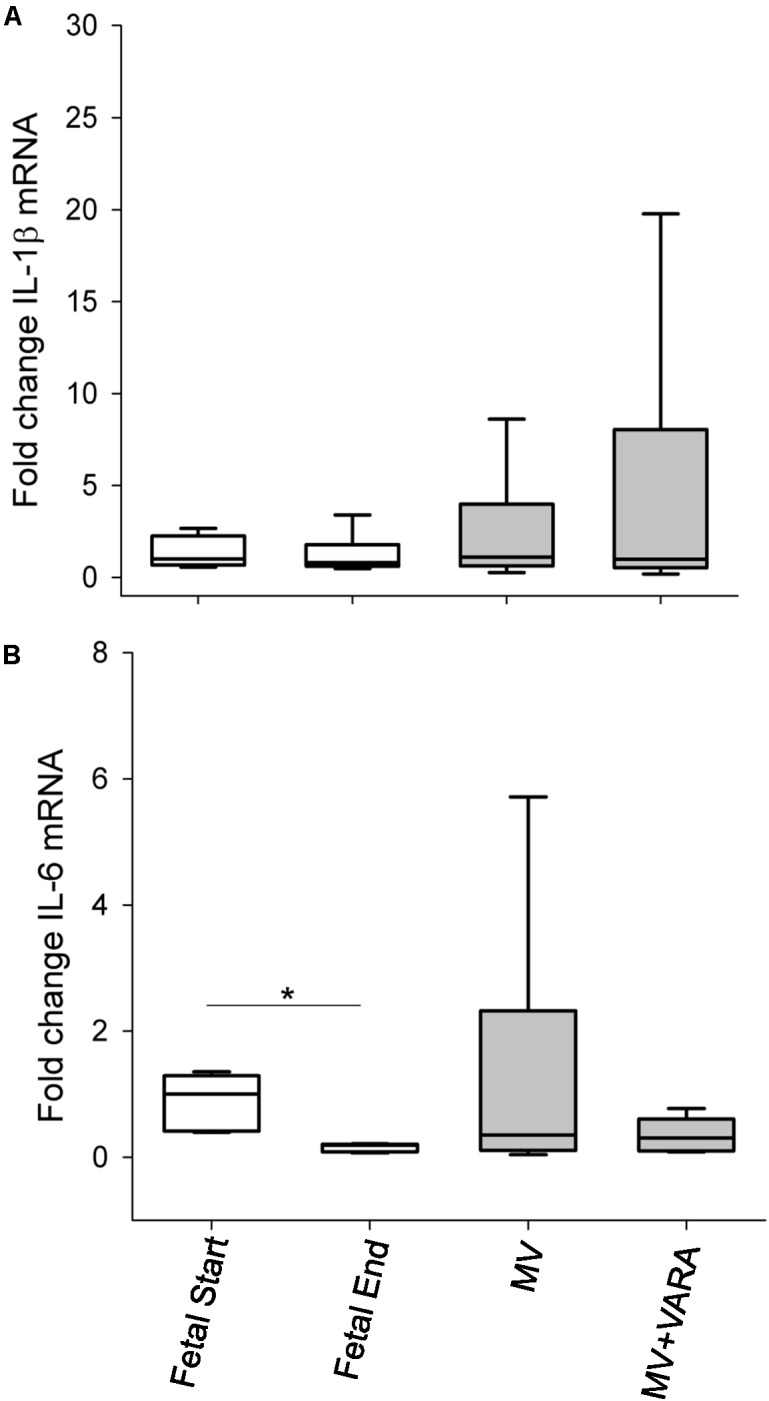
Inflammatory cytokines. Graphs show mRNA level of *IL-1*β **(A)** and *IL-6* **(B)** in the groups of Fetal Start (*n* = 6), Fetal End (*n* = 6), MV (*n* = 7), and MV + VARA (*n* = 6). Values are Median (25th and 75th centile), expressed as fold-change relative to Fetal Start group. Values are Mean (SD). ^∗^indicates *p* < 0.05. MV, mechanical ventilation; VARA, vitamin A and all-*trans* retinoic acid.

mRNA expression levels of the inflammatory markers IL-1β and IL-6 were not different between the MV and MV + VARA groups of preterm lambs (**Figure 5**).

### Oxidative Stress and Antioxidant Gene Expression

**FIGURE 6:**
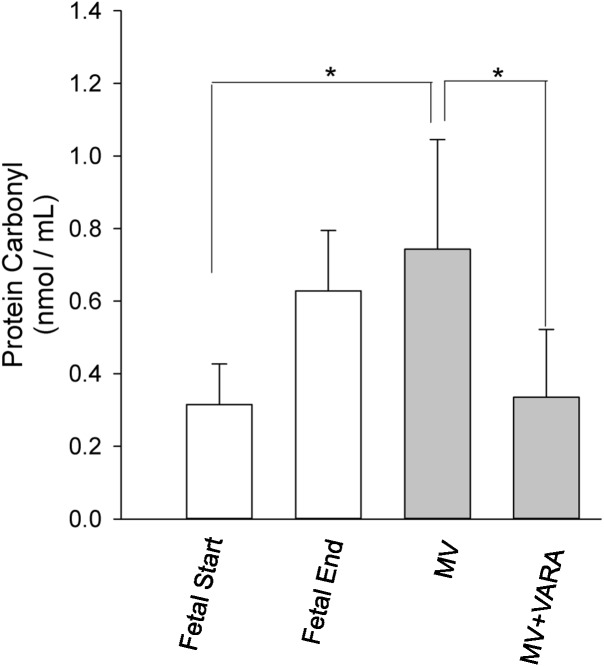
Protein carbonyl abundance. Graphs show protein carbonyl abundance in diaphragm of Fetal Start (*n* = 6), Fetal End (*n* = 7), MV (*n* = 9), and MV + VARA (*n* = 6) groups. Values are Mean (SD). ^∗^indicates *p* < 0.01. MV, mechanical ventilation; VARA, vitamin A and all-*trans* retinoic acid.

Protein carbonyl formation is a measure of oxidative stress and a general indicator of protein oxidation. Diaphragmatic protein carbonyl abundance increased in the MV group compared to the unventilated Fetal Start reference group (*p* < 0.05, **Figure 6**). The increase in abundance of protein carbonyl in mechanically ventilated lambs was prevented by treatment with VARA (*p* < 0.05, **Figure 6**). Changes in protein carbonyl abundance correlated positively with the change observed in UPP activity (*r* = 0.722, *p* < 0.001) and mRNA level of *MuRF1* (*r* = 0.574, *p* < 0.01) (**Supplementary Data Sheet 1**).

**FIGURE 7:**
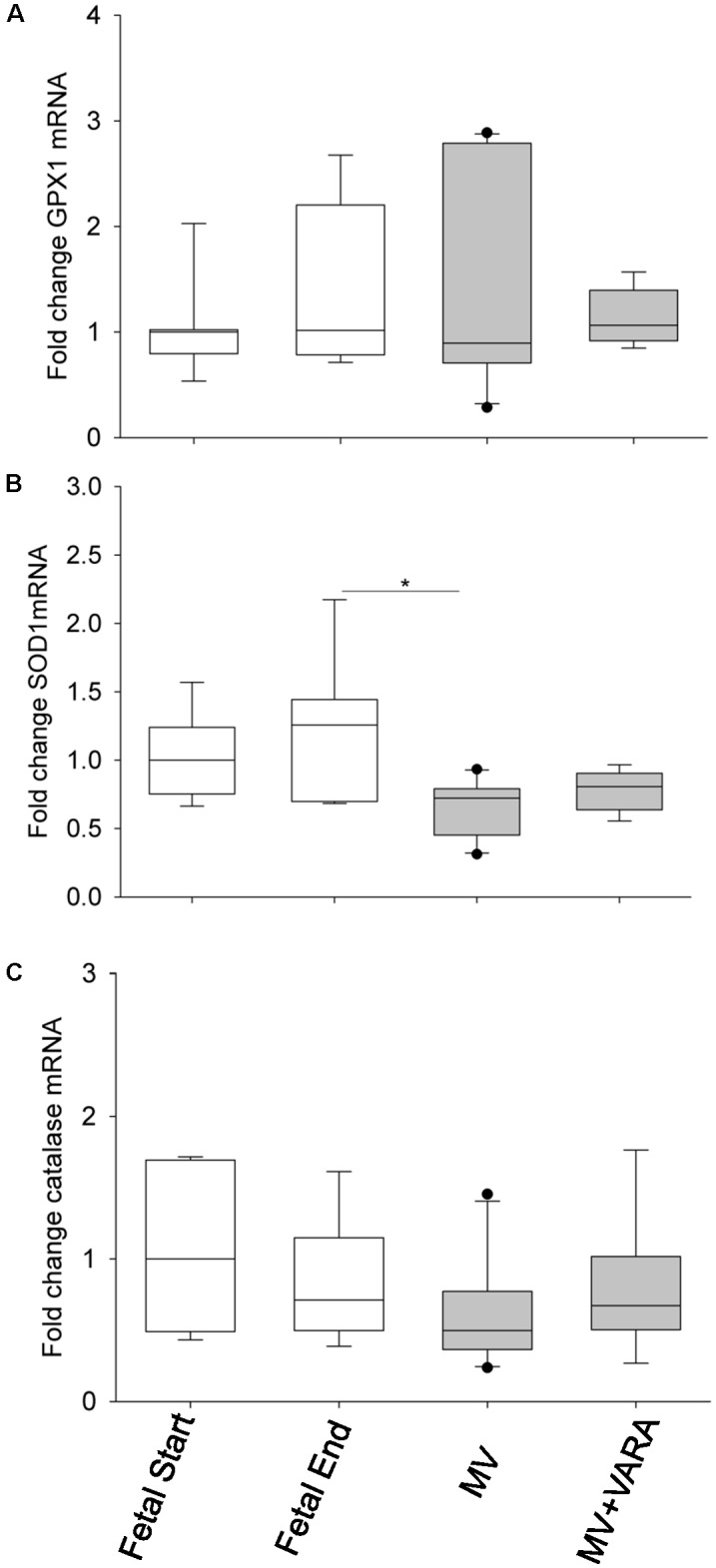
Antioxidant mRNA levels. Graphs show *GPX1* **(A)**, *SOD1* **(B)**, and *catalase* **(C)** mRNA levels in the groups of Fetal Start (*n* = 7), Fetal End (*n* = 7), MV (*n* = 10), and MV + VARA (*n* = 6). Values are Median (25th and 75th centile), expressed as fold-change relative to Fetal Start group. ^∗^indicates *p* < 0.05. GPX1, glutathione peroxidase 1; SOD1, superoxide dismutase 1; MV, mechanical ventilation; VARA, vitamin A and all-*trans* retinoic acid.

We also analyzed mRNA expression levels of three antioxidant genes: glutathione peroxidase 1 (*GPX1)*, superoxide dismutase 1 (*SOD1), and catalase*. *SOD1* mRNA level was significantly lower in the diaphragm of the MV group compared to the Fetal-End unventilated reference group (**Figure 7B**). No effect of MV or treatment with VARA was evident on the mRNA expression levels of *GPX1* (**Figure 7A**) or catalase (**Figure 7C**) genes.

## Discussion

We show that postnatal MV for 3 days disrupts MHC mRNA levels, activates the UPP protein degradation pathway, and promotes oxidative stress in the diaphragm of preterm lambs. Daily VARA treatment during 3 days of mechanical does not affect MHC mRNA expression levels. However, daily treatment with VARA significantly decreases activation of the proteolytic pathway and prevents oxidative stress induced by MV.

These are the first experiments to evaluate the biological effects of MV on molecular signaling in the preterm neonatal diaphragm. Increased proteolysis and oxidative stress in diaphragms of ventilated preterm lambs supports the supposition that preterm infants may be susceptible to VIDD. Remodeling and atrophy of muscle fibers is a key feature of VIDD and accounts for inability to wean adults from MV to unassisted spontaneous breathing (Shanely et al., 2002; Yang et al., 2002). The underlying mechanisms governing muscle fiber changes are probably associated with both pre-translational and post-translational regulations (Yang et al., 2002; Sassoon et al., 2004; Shanely et al., 2004). In adults, diaphragmatic levels of MHC mRNA exhibit little change during the first 18 h of MV (Shanely et al., 2004), whereas MHC mRNA levels are altered over the subsequent 30 h, leading to a slow-to-fast shift in MHC protein expression in the diaphragm (Yang et al., 2002). However, such muscle fiber composition transition cannot be extrapolated simply to the preterm lamb model due to the different composition of muscle fiber type in the immature diaphragm. Our study shows that mRNA expression levels of genes for the predominant fiber types (MHC IIa and I) are significantly lower in the diaphragm of preterm lambs after 3 days of MV compared to unventilated Fetal-End reference lambs, which match the postconceptional age of the preterm lambs after 3 days of ventilation support. Meanwhile, MV reduced *MHC neonatal* mRNA level and increased *MHC IIx* mRNA level. These results need to be interpreted alongside what is known about fiber type development in the lamb diaphragm. Fiber composition analysis in naïve lamb diaphragm at 121 or 127 days of gestation shows that the predominantly expressed isoform is MHC Type IIa (67%) with MHC Type I representing 15% of the total fibers (Cannata et al., 2011; Song et al., 2013a). Subsequently, a decrease in MHC Type IIb/x, and an almost complete loss of neonatal MHC mRNA level occurs from late gestation to term (Cannata et al., 2011). Given the relative proportion of MHC isoforms in the preterm diaphragm, reduced levels of *MHC IIa and MHC I* mRNA after 3 days of MV may contribute to total MHC protein loss. Thus, altered conformations or reduced absolute numbers of contractile proteins may decrease the number of cross-bridges available to generate force, resulting in functional deficit.

The effect of MV on MHC mRNA expression levels may not be the major contributing factor for muscle atrophy in our study. Other studies show that diaphragmatic atrophy and contractile dysfunction occur as early as 12–18 h after onset of MV, accompanied by activation of the proteolytic pathway (Shanely et al., 2002; McClung et al., 2007). Atrophy and contractile dysfunction are exacerbated during longer periods of MV (Shanely et al., 2004; DeRuisseau et al., 2005). Thus, the functional and phenotypic changes in diaphragm are mainly attributable to accelerated protein degradation, along with reduction of anabolic pathways (Shanely et al., 2002, 2004; Powers et al., 2009, 2013), which precedes MHC mRNA change. During MV, diaphragmatic protein breakdown occurs through the degradation of myofibrillar proteins via the ATP-dependent ubiquitin proteasome pathway (UPP). The UPP pathway is regulated transcriptionally through atrophy genes (*MAFbx* and *MuRF1*), as evidenced by the positive association between UPP activity and *MuRF1* mRNA level in the present and previous studies (DeRuisseau et al., 2005). Our data show that 3 days of MV activates the UPP system and induces up-regulation of *MuRF1* mRNA level in our preterm lamb model. Together, these findings support our hypothesis that MV increases protein degradation in the diaphragm of preterm lambs, possibly leading to atrophy and subsequently muscle weakness.

Up-regulation of *MAFbx* and *MuRF1* genes is governed by transcriptional factors FOXO1 and NF-κB during disuse muscle atrophy (Powers et al., 2007; Bonaldo and Sandri, 2013). However, we show that nuclear FOXO1 and NF-κB content remain constant after 3 days of MV. Accordingly, the upstream regulators, Akt signaling and cytokines (IL-1β and IL-6), for FOXO1 and NF-κB, respectively, are not altered in response to MV. We may exclude, therefore, a principal role of the FOXO1 and NF-κB pathways in initiating UPP activation in our preterm MV model. Alternative mechanisms, such as regulation of atrophy-related genes via JNK, AMPK, FOXO3, TRAF6, and Smads, may exist (Bonaldo and Sandri, 2013). Apart from inhibiting nuclear translocation of FOXO, Akt activates its downstream effectors (p70S6K and 4E-BP1) to drive protein synthesis through mTOR. From our analysis of this anabolic signaling cascade, we did not identify an effect of MV on the protein synthesis pathway in this study, although cannot exclude the possibility of a Type II statistical error.

Mechanical ventilation is associated with rapid onset of oxidative stress in the adult diaphragm, occurring within 3–6 h of initiating MV (Zergeroglu et al., 2003). Similarly, our study shows that 3 days of MV increases diaphragmatic protein carbonyl formation by approximately threefold. There is increasing evidence to implicate ROS accumulation with oxidative stress as an atrophic signaling trigger [*i.e.*, ROS activates FOXO and induces atrophic genes, leading to UPP activation (Powers et al., 2011); ROS augments calcium-dependent protease, calpain, and caspase-3 activity (Shanely et al., 2002; Whidden et al., 2010; Powers et al., 2011)]. Independent of accelerated proteolysis, ROS has direct deleterious effects on muscle contractile function by altering myofibrillar Ca^2+^ sensitivity and cross-bridge kinetics (Andrade et al., 2001).

We also aimed to assess the benefit of enteral vitamin A supplementation on the occurrence and severity of VIDD in preterm lambs. We used a combination of vitamin A and all-*trans* retinoic acid (VARA) because this mixture provides substrate for formation of bioactive retinoids, through retinyl esters (Ross et al., 2006a). VARA treatment is effective for reducing severity of bronchopulmonary dysplasia in a hyperoxic rat model and is absorbed via the enteral route, providing an alternative to intramuscular administration. Treatment of rat pups with VARA, for example, increased retinyl esters and improved alveolar formation more than vitamin A alone (Ross et al., 2006b; Ross and Ambalavanan, 2007; Ross and Li, 2007). We used an optimized retinol dosage of 2500 IU/Kg/day, given orogastrically in a 10:1 ratio with all-trans retinoic acid. We based dosage optimization on efficacy to promote alveolar formation, without evidence of elevated liver enzymes. The advantage of orogastric delivery is that it eliminates pain and stress caused by daily intramuscular injection, which is otherwise required for vitamin A supplementation. Retinoic acid, the active metabolite of vitamin A, binds to retinoic acid and retinoic X receptors when transported to the nucleus. These heterodimeric pairs recruit a range of coactivators or corepressors to regulate gene transcription and influence protein production. However, our results show that VARA does not promote transcriptional upregulation of MHC mRNAs during MV.

Our results show that daily enteral VARA attenuates the UPP pathway (lower 20S proteasome activity and *MuRF1* mRNA level), the protein catabolic pathway (lower FOXO1 protein level), and oxidative stress pathway (lower protein carbonyl formation) over 3 days of MV. In particular, our data show that accelerated protein degradation during MV is restored to normal level after VARA. We suggest, therefore, that VARA treatment reduces protein catabolism of the diaphragm of preterm lambs during MV. The decrease in UPP is likely due to down-regulation of atrophic gene *MuRF1* through reduced FOXO1 signaling.

The effect of VARA in preventing VIDD is also supported by the important evidence that VARA treatment reverses ventilator-induced oxidative stress in the diaphragm of preterm lambs. Reduced oxidative stress may explain the decrease in FOXO1 signaling and the resulting decrease in proteolytic activity during VARA treatment. This mechanistic link is supported by other studies (Shanely et al., 2002; Powers et al., 2011) and the significant association between protein carbonyl level and UPP activity/*MuRF1* mRNA level identified in the present study.

Cellular redox potential is maintained by balanced regulation of pro-oxidative and anti-oxidative enzymes. GPX proteins along with SOD and catalase are part of the enzymatic defense system that cells use to reduce free radical-mediated cellular injury. Vitamin A deficiency leads to lower GSH/GSSG ratio in rat liver (Barber et al., 2000), less activity of glutathione-S-transferase in lung (Dogra et al., 1982), and less lung SOD and catalase (Dogra et al., 1983). An antioxidant mechanism of vitamin A may be attributed to transcriptional regulation of antioxidant genes. To test this hypothesis, we examined GPX1, SOD1, and catalase transcripts over the experimental period. Our data show that daily VARA treatment during 3 days of MV does not reduce *GPX1, SOD1*, or *catalase* mRNA levels. However, we should not exclude the possibility that other antioxidant genes are regulated by retinoic acid. For example, recently Haddad and colleagues identified *GPX3* as a primarily retinoid-responsive gene that mediates the antioxidative effects of retinoic acid in human skeletal muscle cell (El Haddad et al., 2012). Alternatively, the antioxidant activity of retinoic acid may be due to the hydrophobic chain of polyene units, which quench singlet oxygen, neutralize thiol radicals, and stabilize and combine with peroxyl radicals (Palace et al., 1999).

Our study has some weaknesses. We note that there is a potential difference in sex distribution between MV + VARA group (constituted of six male lambs) and other groups, although we did not observe a statistical significance due to low sample size. However, preterm male fetuses usually show poorer respiratory, hemodynamic and neurodevelopmental responses than female counterparts. Hence, the male dominance of the MV+VARA group may have reduced the magnitude of the beneficial effect of VARA on reducing the impact of MV on the diaphragm (Willet et al., 1997; Bennet et al., 2007). We also acknowledge that whilst the changes in biochemical and molecular markers of the diaphragm are consistent with damage resulting from MV, we cannot confirm that MV was the cause of this dysfunction. Keeping preterm lambs alive for 3 days without any MV is not achievable using the current model and hence fetal lambs at starting and end gestations were used to account for the effect of maturation. Nonetheless, these fetal lambs have not been exposed to other postnatal managements including nutrition, antibiotics, caffeine and oxygen. Finally the study is limited in that we lack of direct evidence in relation to change of diaphragm function and structure and protein turnover, particularly how passive ventilation stimulates change of contractile properties of the diaphragms in preterm model. Contractile measurements are not feasible due to the geographical separation of the collaborating groups and lack of appropriate muscle function expertise in the preterm lamb laboratory group.

## Conclusion

Three days of MV impairs the preterm diaphragm via abnormal expression of myofibrillar composition, activation of proteases, and oxidative injury. Daily administration of VARA for 3 days attenuates catabolic signaling, probably by protecting cells from oxidative stress. We suggest that enteral VARA may be a promising therapeutic target for maintenance of muscle protein balance in preterm infants at risk of VIDD.

## Availability of Data and Material

The data that support the findings of this study are available from the corresponding author on reasonable request.

## Author Contributions

YS, JP, and KA conceived and design the study. YS performed the laboratory work, analyzed the data, and drafted the manuscript. KA, MD, WL, JA, and CB performed the preterm lamb studies. JP, MD, and KA revised the manuscript. All authors read and approved the final manuscript.

## Conflict of Interest Statement

The authors declare that the research was conducted in the absence of any commercial or financial relationships that could be construed as a potential conflict of interest.

## Abbreviations

BPD: bronchopulmonary dysplasia
GA: gestational age.
GPX1: glutathione peroxidase 1
HSD: honestly significant difference
MHC: myosin heavy chain
MV: mechanical ventilation
p: phosphorylated
PEEP: positive end-expiratory pressure
PIP: peak inspiratory pressure
RA: retinoic acid
ROS: reactive oxygen species
SOD1: superoxide dismutase 1
UPP: ubiquitin proteasome pathway
VARA: vitamin A and all-*trans* retinoic acid
VIDD: ventilator-induced diaphragmatic dysfunction
