# Supplementary material for: Vitamin A Protects the Preterm Lamb Diaphragm Against Adverse Effects of Mechanical Ventilation
Source: Front Physiol. 2018 Aug 13;9:1119. doi: 10.3389/fphys.2018.01119 (PMC6099107; doi:10.3389/fphys.2018.01119)
Supplement: Supplementary file 1 [file Data_Sheet_1.PDF]

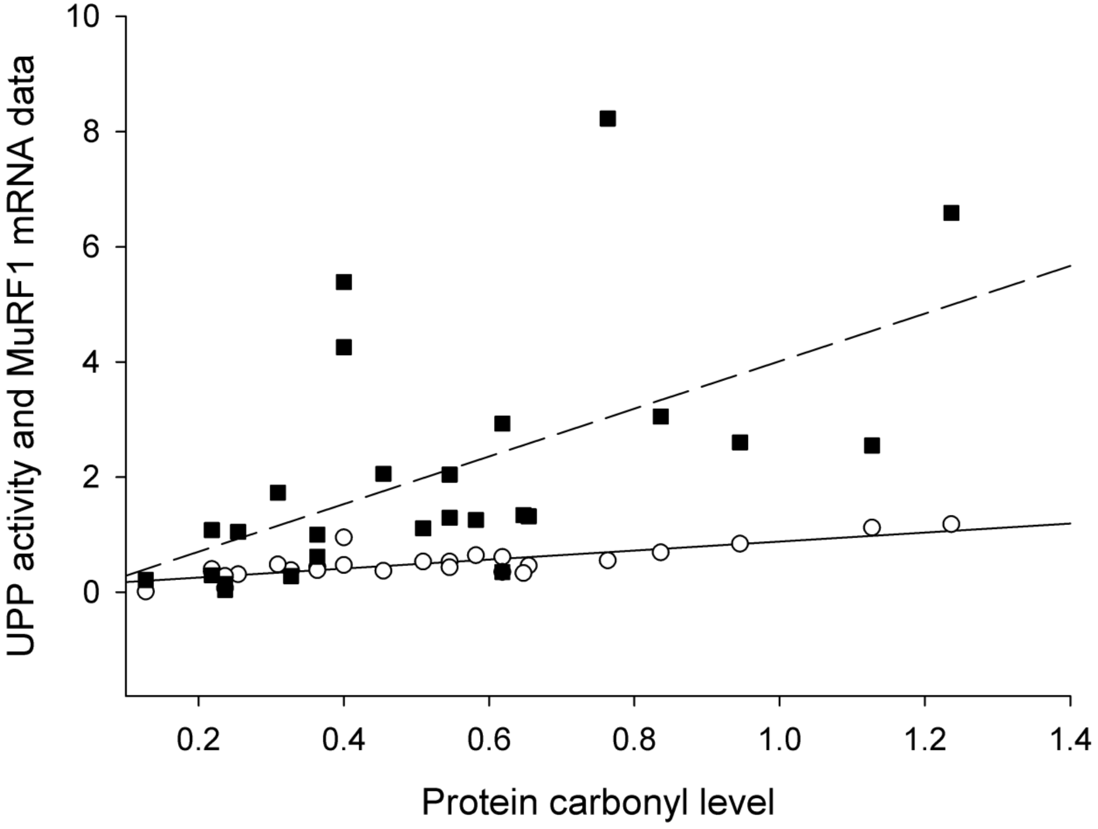

Supplementary Figure 1. Association of Protein Carbonyl Abundance with UPP Activity / *MuRF1* mRNA Level: A linear relationship is established between protein carbonyl level and UPP activity (open circle, solid line) / *MuRF1* mRNA amount (closed square, dashed line). UPP: ubiquitin proteasome pathway

Additional file 1

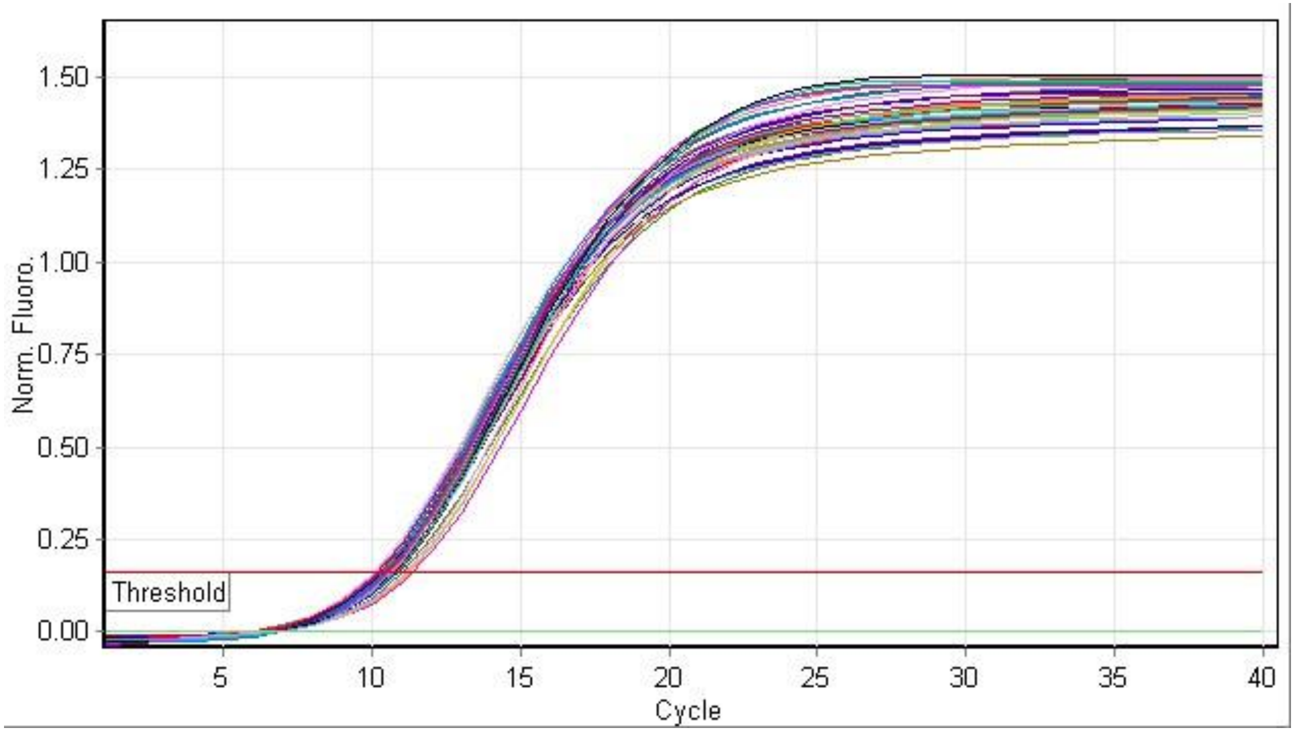

Supplementary Figure 2. 18S RNA qPCR: A similar CT value was obtained for 18S RNA for all lambs across all study groups showing suitability of 18S as a house-keeping gene.
